# Knockout and Inhibition of Ape1: Roles of Ape1 in Base Excision DNA Repair and Modulation of Gene Expression

**DOI:** 10.3390/antiox11091817

**Published:** 2022-09-15

**Authors:** Zhouyiyuan Xue, Bruce Demple

**Affiliations:** 1 Department of Pharmacological Sciences, Renaissance School of Medicine, Stony Brook University, Stony Brook, NY 11794-8651, USA; 2 Molecular and Cellular Biochemistry Program, Stony Brook University, Stony Brook, NY 11794-8651, USA

**Keywords:** Ape1, *APEX1*, base excision repair, redox, Ape1 inhibitor, knockout, CH12F3, HEK293 FT, Compound **3**, APX2009

## Abstract

Apurinic/apyrimidinic endonuclease 1/redox effector-1 (Ape1/Ref-1) is the major apurinic/apyrimidinic (AP) endonuclease in mammalian cells. It functions mainly in the base excision repair pathway to create a suitable substrate for DNA polymerases. Human Ape1 protein can activate some transcription factors to varying degrees, dependent on its N-terminal, unstructured domain, and some of the cysteines within it, apparently via a redox mechanism in some cases. Many cancer studies also suggest that Ape1 has potential for prognosis in terms of the protein level or intracellular localization. While homozygous disruption of the Ape1 structural gene *APEX1* in mice causes embryonic lethality, and most studies in cell culture indicate that the expression of Ape1 is essential, some recent studies reported the isolation of viable *APEX1* knockout cells with only mild phenotypes. It has not been established by what mechanism the Ape1-null cell lines cope with the endogenous DNA damage that the enzyme normally handles. We review the enzymatic and other activities of Ape1 and the recent studies of the properties of the *APEX1* knockout lines. The *APEX1* deletions in CH12F3 and HEK293 FT provide an opportunity to test for possible off-target effects of Ape1 inhibition. For this work, we tested the Ape1 endonuclease inhibitor Compound **3** and the redox inhibitor APX2009. Our results confirmed that both *APEX1* knockout cell lines are modestly more sensitive to killing by an alkylating agent than their Ape1-proficient cells. Surprisingly, the knockout lines showed equal sensitivity to direct killing by either inhibitor, despite the lack of the target protein. Moreover, the CH12F3 *APEX1* knockout was even more sensitive to Compound **3** than its *APEX1^+^* counterpart. Thus, it appears that both Compound **3** and APX2009 have off-target effects. In cases where this issue may be important, it is advisable that more specific endpoints than cell survival be tested for establishing mechanism.

## 1. Introduction

Genomic DNA is under the relentless threat of molecular decay due to endogenous and exogenous damage, which threatens genetic stability. Reactive oxygen species (ROS), notably the byproducts of aerobic metabolism, produce an array of DNA lesions, including 8-oxoguanine (8-oxoG) and thymine glycol. Hydrolysis and various other reactive metabolites add many other DNA lesions to this burden [1]. Many of these lesions disrupt DNA structure only slightly or not at all, but they nonetheless can exert mutagenic or cytotoxic effects if left unrepaired. Ring-opened aldehyde apurinic/apyrimidinic (AP) sites can form a covalent bond with neighboring nucleotides or histone lysines, which can result in highly toxic DNA interstrand cross-links and histone-DNA cross-links [2,3].

Base excision DNA repair (BER) is the frontline process that deals with most of the small lesions. Following the removal of a damaged base by a DNA glycosylase, the 5′-phosphodiester bond of the resulting apurinic/apyrimidinic (AP) site is cleaved by Ape1; that enzyme also cleaves spontaneous hydrolytic AP sites. The gap-filling reaction is finished by other BER proteins. Ape1 is also reported to be involved in activating some transcription factors by maintaining their reduced forms; for this, the protein is also called Redox effector-1 (Ref-1). This role may contribute to cellular defenses against oxidative stress. For example, Ape1 can induce the tetramerization and expression of transcription factor p53, which can then upregulate the expression of superoxide dismutase 2 [4]. However, the detailed mechanism by which Ape1 modulates transcription factors remains unclear. A current model is via thiol exchange, with one Ape1 cysteine attacking a disulfide bond in the target protein, eventually resolving to yield the reduced target protein (and oxidized Ape1). Cysteine-65 of Ape1 has been implicated in this process, despite its being sequestered inside the protein structure [5].

Despite broad interest in the roles of Ape1 in BER and transcriptional control, animal studies have been limited because the *APEX1* gene is essential and knockouts are embryonic-lethal. *APEX1* conditional knockout mice have been generated by the Cre/lox method, but they still retain considerable amounts of Ape1 in some organs even after the knockout system is activated. Mice haploinsufficient for Ape1 are more sensitive to oxidative stress and have a higher chance of developing adenocarcinoma and lymphoma [6]. Surprisingly, a human *APEX1* transgene could not compensate for the murine *APEX1* knockout (*APEX1*-KO) during mouse embryo development, suggesting the importance of dynamic Ape1 expression regulation [7]. This review will summarize the biological function of Ape1 in the order of its activities based on knockout studies, and we will assess the prospects for Ape1 as a target for cancer therapy.

## 2. Ape1 Overview

### 2.1. Base Excision Repair and DNA End Processing

BER corrects much of the oxidatively-induced and other endogenous base damage in most organisms, including humans. The first step of BER is the removal of the damaged base by a DNA glycosylase, which results in an AP site. If the DNA glycosylase is monofunctional, in mammalian cells the AP site will first be processed by Ape1. Ape1 hydrolyzes the 5′ phosphodiester bond at the AP site, leaving a normal 3′-OH nucleotide on one side and a 5′-deoxyribose-5-phosphate (5′-dRP) on the other. Some DNA glycosylases are bifunctional, able to further process the AP site through a β-lyase activity (e.g., OGG1) or a β,δ-lyase activity (e.g., NEIL1). β-elimination produces a 3′-terminal unsaturated hydroxyaldehyde, which must be removed by Ape1 to allow DNA synthesis, and a normal 5′-phosphate. β,δ-elimination leaves a 3′-phosphate and a 5′-phosphate, bracketing a small gap. In that case, polynucleotide kinase phosphatase (PNKP) provides a DNA synthesis primer by removing the 3′-phosphate, which Ape1 does very inefficiently [1]. The next step is gap filling. During short patch BER (SP-BER), Ape1, Polβ, X-ray repair cross-complementing protein 1 (Xrcc1) in complex with DNA ligase IIIα (Lig3), and Poly(ADP-ribose) polymerase (PARP) are consecutively recruited to the lesion for repair. Polβ also removes the 5′-dRP by using a dRP lyase activity in its separable N-terminal domain, with the polymerase inserting a nucleotide to replace the damaged one. Finally, Lig3 can seal the nick. Proliferating cell nuclear antigen (PCNA), Ape1, Polβ (or DNA polymerase δ/ε in proliferating cells), DNA ligase I (Lig1), PARP, and Flap endonuclease 1 (Fen1) work together to conduct long patch BER (LP-BER), which involves strand replacement DNA synthesis. The resulting flap is cleaved by Fen1 and the nick sealed by Lig1.

Ape1 uses the same active site to conduct exonuclease and endonuclease activity. For correctly paired nucleotides, the Ape1 exonuclease activity is only ~1% of the AP endonuclease activity; for mismatched nucleotides the activity can be up to 10% of the AP endonuclease. The Ape1 exonuclease may contribute to the fidelity of BER, since Polβ lacks intrinsic or other associated 3′-exonuclease proofreading activity, with an error rate around 10^−4^ (vs. 10^−6^ or better for replication polymerases) [8]. Ape1 exonuclease activity can act on a variety of other substrates, including the nucleoside analog β-l-dioxolane-cytidine and a biotinylated nucleotide [9,10]. However, Ape1′s exonuclease activity varies on different substrate structures. For example, the Ape1 exonuclease activity is lower for blunt-end substrates than for substrates with recessed 3′-ends [10]. It is worth mentioning that Ape1 cannot excise nucleotides from substrates containing more than one mismatched 3′-residue.

Ape1 acts as an end processor in single-strand break repair. Single-strand breaks (SSB), which can result from free radical attack or spontaneous β-elimination at an AP site, is another prevalent type of lesion. Unlike the SSB generated during BER, these SSB are not sheltered by proteins and they need to be end-processed before DNA polymerases and DNA ligases can act [11]. Although most bulky adducts and double helix-distorting lesions are repaired by nucleotide excision repair, that pathway is unable to remove lesions at the end of single-strand breaks or double-strand breaks. Ape1 can excise bulky lesions on recessed 5′ ends, blunt ends, and 3′ overhangs shorter than two nucleotides [12]. In addition, Lin et al., pointed out that Ape1 can initiate 3′ end resection on SSB and is indispensable for inducing the ATR-Chk1 signaling pathway in Xenopus extracts [13]. The homologous Ape2 protein has much weaker AP endonuclease activity than does Ape1, but Ape2 operates to remove certain types of blocked DNA 3′ termini [14]

### 2.2. Redox Signaling and Oxidative G-Quadruplex Formation in Gene Expression

Genome instability, resistance to cell death, senescence, and angiogenesis are some of the hallmarks of cancer, and Ape1 may function in most of these processes. A meta-analysis study concluded that a high Ape1 expression level is associated with a short overall survival rate of patients with solid tumors [15]. This seems paradoxical because the primary role of Ape1 seems to be helping maintain genetic stability by repairing damaged DNA and inducing cell apoptosis by activating p53. However, some cancer cells may hijack this process to resist DNA damage induced by chemotherapy. Part of the reason for the upregulation of Ape1 could relate to the high ROS level in cancer cells [16]. For example, knockdown of Ape1 by siRNA in B-lymphoblastoid TK6 cells and colon tumor HCT116 cells dramatically sensitized the cells to killing by the antitumor drug bleomycin or to X-ray treatment [17].

Ape1 has been reported to activate various transcription factors, including AP-1, HIF-1α, and NF-κB. The mechanism of this activation is thought to be via a redox reaction [18,19,20]. According to the Ape1 co-crystal structure with DNA [21], no disulfide bond can form between any two cysteine residues. Homology analysis revealed that, except for Cys 65, which is unique to mammals, the rest of the cysteines are conserved among vertebrates. Single cysteine-to-alanine mutations revealed that only C65A abolished Ape1-induced AP-1 DNA binding [22,23]. In addition, cysteine substitution of zebrafish Ape1 Thr58, equivalent to Cys65 in mammalian Ape1, conferred redox activity on the zebrafish protein expressed in human cells [24]. Further cysteine-to-alanine substitutions showed that Ape1 C93A/C99A double mutations lost redox activity and Ape1 with all cysteines substituted except Cys65, Cys93, and Cys99 retained the redox activity [25]. Two other studies [20,26] found that Cys65 (Cys64 in murine Ape1) was unnecessary for the Ape1 redox activity.

Ape1 is also proposed to modulate transcription by stabilizing G-quadruplexes in certain promotors. Guanine is the DNA base most susceptible to oxidative damage due to its low redox potential [27]. Therefore, lesions such as 8-oxoG can form throughout the genome when ROS accumulates. Within a gene body, 8-oxoG can stall transcription [28,29]. However, 8-oxoG in a promotor can induce the formation of BER-stabilized G-quadruplexes that enhance gene expression [30]. In this scenario, Ogg1 first excises the 8-oxoG base and hands the AP repair intermediate to Ape1. The AP site and BER machinery destabilize the normal double helix structure and lower the energy barrier for forming G-quadruplexes in some sequences [31]. The AP endonuclease of Ape1 is highly attenuated at abasic sites in certain parts of a G-quadruplex structure, compared with its activity on double-strand DNA [32]. In such cases, rather than incising the phosphodiester bond, Ape1 now helps stabilize these G-quadruplexes. The natively unstructured Ape1 N-terminus is essential for this type of binding, with the acetylation of N-terminal lysines fine-tuning the residence time of bound Ape1 [33]. Recent studies found that some key oncogenes, including *HIF-1α*, *VEGF,* and *c-MYC*, have at least one G-quadruplex in their promoter [34,35,36]. These dual and overlapping functions (repair and gene expression) highlight the potential of Ape1 as a cancer therapy target. Ape1 inhibitors that separately target either the redox activity or the nuclease activity are available, some of them now undergoing clinical trials, which will be discussed in a later section.

### 2.3. RNA Processing

RNA accounts for 80–90% of a cell’s total nucleic acid and is more vulnerable to damage than is DNA [37]. All the same sources of damage mentioned earlier for DNA also act on RNA, which is further susceptible to many nucleases in the cell. Damaged gene-coding mRNA can produce truncated or misfolded proteins, while damaged non-coding mRNAs can compromise gene regulation [38,39].

AP endonuclease activity for RNA and the ribonuclease H activity were first observed for Ape1 in the context of lesion-containing DNA/RNA hybrids [40]. In addition, single-strand RNA endonuclease, weak 3′-phosphatase, and 3′-exoribonuclease activities have also been reported for Ape1, and they depend on the same active site as the DNA-cleaving nuclease [40,41]. Therefore, impairing the RNase H activity without disturbing the AP endonuclease activity is a significant barrier to determining the biological relevance of these activities.

Lee et al., first reported that Ape1 could cleave c-Myc rRNA between UA and CA in single-strand or weakly paired regions to control the amount of the transcript [42,43]. Tell et al., [44] found that Ape1, through its N-terminal domain, physically interacts with nucleophosmin (Npm1) and colocalizes in the nucleolus. This interaction is disrupted by the acetylation of some N-terminal Ape1 lysine residues, which releases additional enzyme into the rest of the nucleus, suggesting that this response allows a rapid deployment of BER in the face of DNA damage [44]. Suppression of Ape1 results in the accumulation of oxidized RNA and reduced protein synthesis [45]. Although Ape1 lacks the canonical mitochondrial targeting signal, it has been found in mitochondria [45] and protects the organelle’s DNA and RNA [46,47,48].

### 2.4. Ape1 Knockout Cell Lines: How Do They Survive?

The biological function of Ape1 has been studied using genetic knockout and RNAi-mediated knockdown approaches. Independent studies in two different mouse strains both showed that the homozygous deletion of murine *APEX1* leads to embryonic degeneration after implantation, in one case by day 5.5 and in others by day 9.5 [49,50,51], with degeneration occurring throughout the embryo. However, these studies merely indicated the critical importance of Ape1 without shedding light on which of its activities might be the essential one(s).

Recently, two *APEX1* conditional knockout mouse models were developed to study the relationship between Ape1 and senescence or nervous system development. A tissue-specific *APEX1*-KO in the central and peripheral nervous system did not discernibly affect embryonic development and pups were born in Mendelian ratios [51]. However, the rapid accumulation of DNA damage, accompanied by neuronal degeneration, was observed soon after birth, with all the animals dead by 3 weeks of age [51]. Li et al., [52] induced *APEX1*-KO in all tissues using an inducible Cre/lox system. Mice that lost *APEX1* earlier (day 7 or day 12 after birth) exhibited severe dysfunction phenotypes, including impaired growth, reduced organ size, and premature senescence, with more than 80% of the animals dying before day 28. Mice that lost *APEX1* later (6 weeks) exhibited milder defects but did show clear signs of premature aging by 8 months [52]. Although both studies failed to eliminate Ape1 protein completely, they indicated that the effects of Ape1 deficiency vary in different developmental stages and organs.

Generally, *APEX1* haploinsufficiency or knockdown phenotypes are similar to but milder than those with *APEX1* homozygous knockouts. Heterozygous *APEX1*-KO mice showed an increased sensitivity to DNA-damaging agents (e.g., ultraviolet-B radiation, 2-nitropropane, and azoxymethane) and redox dysregulation, accompanied by an increased frequency of spontaneous tumors [6,53,54,55]. Given the limitations of animal studies, efforts to identify the Ape1 activities required for survival have been addressed by manipulating cells in culture. Two independent studies confirmed the vital role of the Ape1 endonuclease in a mouse line [7] and in multiple human cell lines [56], with the cysteine-64/65 residue being unnecessary for cell survival. Suppression of Ape1 via siRNA in HCT116 (colon tumor), MCF7 (breast tumor), or TK6 (normal but immortalized human lymphoblasts) led to cell cycle arrest within 48 h, accompanied by the accumulation of abasic damage in genomic DNA and the activation of apoptosis [56]. These defects were all reversed by the expression of the non-homologous and structurally unrelated yeast Apn1 protein, providing very clear confirmation of the importance of supporting BER. Izumi et al., used vectors expressing different mutant Ape1 proteins in cells expressing an inducible *APEX1*-KO system. The results showed that the expression of Ape1 lacking the redox activity rescued the apoptotic phenotype, while nuclease-defective Ape1 or Ape1 mutated in Lys6 and Lys7 did not [7].

Interestingly, emerging studies reported that partial Ape1 deficiency did not detectably affect the replication of HeLa cells, CH12F3 cells, HEK293 FT cells, HCT116 cells, and HCC1937 cells [57,58,59,60]. The first report of a viable *APEX1*-KO was in a study addressing the role of Ape1 in AID-mediated class switch recombination in Ig genes [61], in which a rescue vector expressing Ape1 was maintained while all 3 *APEX1* copies in the hypotriploid mouse line CH12F3 were deleted. Surprisingly, removal of the rescue plasmid did not affect cell proliferation, although the complete *APEX1*-KO did sensitize cells to the simple alkylating agent methylmethane sulfonate (MMS) [61]. Recently, a total knockout of *APEX1* in (human) HEK293 FT cells (which also have three copies of the gene) gave a very similar result to that of CH12F3 cells; there was no impact on cell proliferation and only a modest increase in MMS sensitivity [59]. A severe Ape1 knockdown leads to more unrepaired AP sites in genomic DNA [56], but the mRNA levels of both monofunctional DNA glycosylases (e.g., MutyH, Ung) and multifunctional DNA glycosylases (e.g., Ogg1 and Neil2) were not significantly changed [54,59,62]. Thus, DNA glycosylase expression was not changed to compensate for the Ape1 deficiency and the extra unrepaired AP sites most likely reflect sluggish BER, with an accumulation of this intermediate. In this context, Ape1-deficient cells might have increased tolerance of AP sites in the genome. Another possibility is that other DNA repair pathways are elevated as a compensating mechanism for the loss of Ape1.

### 2.5. Off-Target Effects in Cell Killing by Ape1 Inhibitors

Inhibitors are simple and effective tools with which to study the biological functions of a protein, although their effectiveness can be limited by their targeting specificity. For Ape1, inhibitors have been important reagents in efforts to distinguish which of its activities underlies which phenotype. Inhibitors have been developed with specificity for either the nuclease [63,64,65,66,67] (Table 1) or the redox activity [23,68,69,70,71] (Table 2). Moreover, an inhibitor of the Ape1 redox activity is now also in clinical trials for a rare ocular disease [69]. While it may not matter for the clinical effectiveness of current applications, off-target effects of inhibitors could produce misleading conclusions during further drug development. As best we can tell from the published literature, the Ape1 inhibitors have not been thoroughly tested for possible off-target effects. Here we list some of the widely used Ape1 inhibitors and summarize the data on their possible off-target effects (Table 1 and Table 2).

## 3. Materials and Methods

### 3.1. MTT Cell Viability Assay

HEK293 FT wild-type and *APEX1*-KO cells were plated into 96-well plates at a density of 1.3 × 10^4^ cells/well in DMEM with 10% fetal bovine serum (FBS) and antibiotic/antimycotic mix (Sigma, A5955-100, St. Louis, MI, USA). CH12F3 wild-type and *APEX1*-KO cells were collected by centrifugation and resuspended in phenol red-free RPMI-1640 with L-glutamine, 10% FBS, and antibiotic/antimycotic mix (Sigma, A5955-100, St. Louis, MI, USA) and then plated on 96-well plates at a density of 2.2 × 10^4^ cells/well. The plates were incubated for 24 h at 37 °C in a 5% CO_2_ humidified incubator. Immediately prior to use, the Ape1 inhibitors and methylmethane sulfonate (MMS) were diluted with a culture medium containing 3% FBS. For both HEK293 FT and CH12F3 cells, the FBS in the medium was reduced from 10% to 3% before the addition of inhibitors or MMS. After 24 h of treatment, MTT (3-(4,5-dethylthiazol-2-yl)-2,5-diphenyltetrazolium bromide) was then added at a concentration of 0.5 mg/mL, followed by a 3-h incubation. Stop solution (1% SDS and 0.01 M HCl) was used to dissolve formazan crystals, and the absorbance was measured at 590 nm.

### 3.2. Western Blotting

Cells were collected by scraping (HEK293 FT) or centrifugation (CH12F3) and lysed in radioimmunoprecipitation assay (RIPA) buffer (150 mM NaCl, 1% NP-40, 0.5% sodium deoxycholate, 0.1% sodium dodecyl sulfate (SDS), 50 mM Tris-HCl, pH = 8.0, and complete protease inhibitor cocktail (Roche, 11697498001, Indianapolis, IN, USA). An aliquot of each protein sample was used to determine the protein concentration by Bradford assay (Bio-Rad, 5000006, Hercules, CA, USA). The remainder of the protein samples were boiled in Laemmli buffer (2% SDS, 5% 2-mercaptoethanol, 10% glycerol, 0.01% bromophenol blue, and 0.062 M Tris-HCl, pH 6.7) for 5 min and diluted to a protein concentration of 1 mg/mL. Protein samples were resolved in 10% SDS- polyacrylamide gels according to the Thermo-Fisher gel casting protocol (Thermo-Fisher Document Part 0909.INS, Waltham, MA, USA) and transferred to hydrophilic polyvinylidene fluoride membranes (Sigma, IPFL20200, St. Louis, MI, USA). The membranes were blocked in 5% non-fat milk for 1 h at room temperature and incubated with rabbit polyclonal anti-Ape1 antibody (1:800, Novus, #NB100-101SS, Centennial, CA, USA) and rabbit polyclonal anti-GAPDH antibody (1:1,000, Rockland, 600-401-A33, Limerick, PA, USA) at 4 °C overnight. The blots were then washed with blocking buffer and incubated with IRDye 800CW labeled goat anti-rabbit IgG (1:10,000, LI-COR, 926-32211, Lincoln, NE, USA) at room temperature for 30 min before imaging on a LI-COR Odyssey.

## 4. Evaluation of Compound 3 and APX2009 for Possible Off-Target Effects

**Figure 1:**
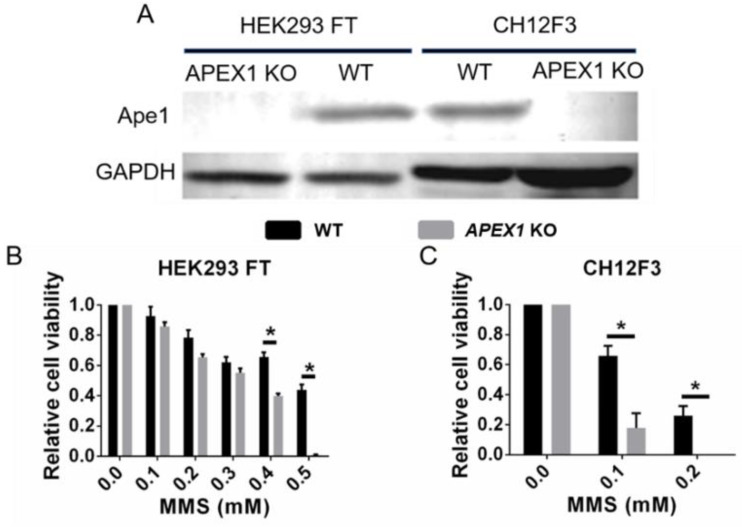
Ape1 deficiency sensitizes HEK293 FT and CH12F3 cells to methylmethane sulfonate (MMS). (**A**) Ape1 protein levels in HEK293 FT wild-type, HEK293 FT *APEX1*-KO, CH12F3 wild-type, and CH12F3 *APEX1*-KO cells. Cell viability of HEK293 FT wild-type and HEK293 FT *APEX1*-KO (**B**) and CH12F3 wild-type and CH12F3 *APEX1*-KO (**C**) cells after 24 h treatment with the indicated amount of MMS. Cell viability was determined using the MTT assay. * *p* < 0.01. Data are presented as the mean ± SD, with *n* = 4 per group, and analyzed by Student *t*-test.

We addressed the question of Ape1 inhibitor targeting by employing the two published *APEX1*-KO cell lines—mouse CH12F3 [61] and human HEK293 FT [59]—both of which appear to function as well without Ape1 protein as they do with it. We verified the *APEX1*-KO lines by Western blotting, which confirmed the absence of Ape1 protein (Figure 1A). We also confirmed that, compared with the parental lines, both types of *APEX1*-KO cells had increased sensitivity to cell killing by MMS, noting that CH12F3 was inherently more MMS-sensitive than HEK293 FT (Figure 1B,C).

**Figure 2:**
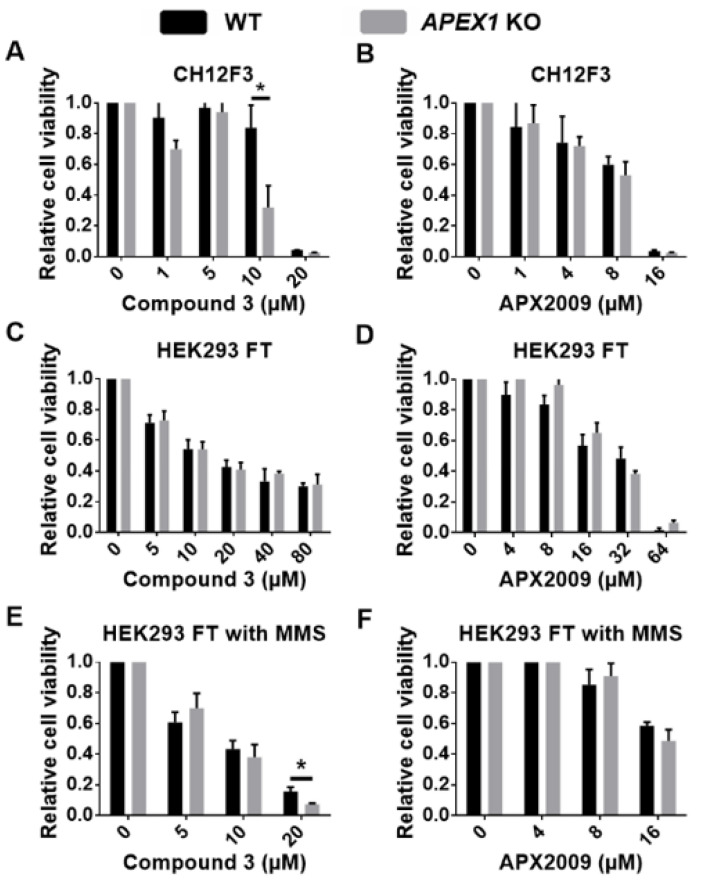
Direct cell killing and off-target effects of Compound **3** or APX2009. CH12F3 *APEX1*^+^ cells and *APEX1*-KO cells were treated for 24 h with the indicated amounts of Compound **3** (**A**) or APX2009 (**B**). HEK293 FT cells were treated with compound **3** (**C**) or APX2009 (**D**) alone or combined with 0.2 mM MMS (**E**,**F**). Cell viability was determined using the MTT assay. * *p* < 0.01. Data are presented as mean ± SD, with n = 4 per group, and analyzed by Student *t*-test.

Following this confirmation of their Ape1 status, we measured these cell lines for the direct toxicity of Ape1 inhibitors that target two different functions: the AP endonuclease inhibitor Compound **3** [63] and the redox inhibitor APX2009 [68]. These agents can kill Ape1-proficient cells; therefore, if they are highly targeted, the expectation was that this toxicity would be lost in the absence of Ape1. Instead, CH12F3 *APEX1*-KO cells exhibited even greater sensitivity to direct killing by Compound **3** than did their *APEX1^+^* counterparts, but with a seemingly narrow window around 10 µM inhibitor treatment (Figure 2A). For the APX2009 inhibitor, we observed no *APEX1*-dependent difference for CH12F3 killing (Figure 2B).

For HEK293 FT cells, the survival of the *APEX1^+^* and *APEX1*-KO versions was the same under all concentrations tested for both Compound **3** and APX2009 (Figure 2C,D). Thus, it appears that the direct killing of cells by either Compound **3** or APX2009 includes substantial off-target effects.

To test whether the reduction of cell viability resulted from insufficient BER, we used a combined treatment of HEK293 FT cells with 0.2 mM MMS and either of the inhibitors. MMS generates numerous base lesions that are processed by BER. The Compound **3** plus MMS treatment reduced the viability of wild-type and *APEX1*-KO cells to a similar extent for Compound **3** concentrations up to 10 μM (Figure 2E). That result is compatible with inhibition of Ape1 activity in vivo [67], with the Compound **3**-treated *APE1^+^* cells being a phenocopy of *APE1*-KO cells. Note that there was cytotoxic synergy between Compound **3** and MMS (compare Figure 2C,E). The biological significance of the difference seen at 20 μM Compound **3** combined with MMS is hard to judge, as the survival of the Ape1-expressing cells was already quite low (Figure 2E). Because APX2009 is an inhibitor of the Ape1 redox activity rather than the nuclease, we expected that it would not sensitize *APEX1^+^* cells to MMS, which was the case, and with no additional effect in the *APEX1*-KO cells (compare Figure 2D,F). Kelley et al., [68] reported that APX2009 enhanced Ape1 DNA repair activity in human fibroblasts and rodent neuronal cells; however, we did not find evidence for this effect in HEK293 FT cells.

## 5. Discussion: Mechanism of Off-Target Effects of Ape1 Inhibitors

The Ape1 inhibitors examined in our work were developed based on their ability to suppress specific Ape1 activities [63,68]. For their potential as cancer therapy agents, cell killing was explored as a biological endpoint, although that is inherently a crude readout of toxic effects, because numerous pathways can lead to that single endpoint. While the effects of the nuclease inhibitor Compound **3** in Ape1-expressing cells were consistent with its interference with DNA repair (sensitization to MMS), it was unexpected that the agent’s direct killing effects were essentially independent of the cellular Ape1 status.

Given the verified importance of Ape1 in routine DNA maintenance [51,52,56,72], the surprising viability of the two *APEX1*-KO lines tested here could reflect in those cases the mobilization of other repair enzymes or pathways. For cells undergoing DNA replication, as in our experiments, the accumulation of unrepaired AP sites would lead to blocked replication forks, with a constant need for the activation of fork protection pathways to prevent the formation of double-strand breaks [73]. Thus, it is possible that, in addition to their known targeting of Ape1, Compound **3** and perhaps APX2009 also interfere with the fork protection systems. Another possibility is that the accumulation of such unrepaired AP residues leads to the formation of some sites that have other lesions positioned nearby (clustered lesions [74,75,76]), which could require the participation of additional repair systems. However, such explanations do not account very well for the lack of difference in toxicity between the *APEX1^+^* and the *APEX1*-KO lines.

Clearly, future work on this issue will have to center on determining whether additional DNA repair pathways are indeed marshalled in Ape1-deficient cells to enable their survival. Alternative possibilities include the suppression of apoptosis pathways or other cell death mechanisms. Furthermore, the off-target effects of the inhibitors may actually have advantages in tumor treatment, provided that cancer cells are shown to be more sensitive to them than normal cells in the same tissues. Indeed, such off-target effects could even help identify new pathways of cell survival in the face of DNA damage.

## 6. Concluding Points

- Discovered as a DNA repair enzyme, Ape1 has been associated with multiple other roles, including both redox and non-redox activation of transcription factors;
- Ape1 can stabilize G-quadruplexes by binding but not cleaving AP sites in certain positions, which can mediate some transcriptional effects;
- Ape1 is essential for embryonic development in mice and probably for mammals in general;
- Genetic knockdown and knockout experiments indicate that the DNA repair function is essential in most cell types in culture;
- Inhibitors have been developed to target either the nuclease activity of Ape1 or its redox activity;
- Two viable cell lines have been developed with the Ape1-coding gene *APEX1* deleted; these lines have mild phenotypes, the basis of which is unknown;
- The Ape1 inhibitors show similar toxic effects in *APEX1*-knockout cells and their *APEX1^+^* counterparts, indicating that the compounds have significant off-target effects.

## Figures and Tables

**Table 1:** Major Ape1 endonuclease inhibitors and their characteristics.

Inhibitor Name	Ape1 AP Endonuclease Assay	AP Site Reactivity of Compound	Ape1 Redox Activity	Other Ape1 Activity	Other DNA Repair Pathways
**Compound 3** [63]	HeLa WCE * incision assay	NA *	NA	Ape1 AP site binding not affected	NA
**Lucanthone** [64,65]	U251-MG glioblastoma multiforme cell WCE incision assay	Enzyme digestion assay; no binding	No effect	Did not affect exonuclease activity	NA
**CRT0044876** [66]	Recombinant Ape1 incision assay	Enzyme digestion assay; no binding	NA	3′-phosphatase and 3′-phosphoglycolate diesterase activities not affected	Did not potentiate the cytotoxicity of ionizing radiation or UV light
**AR03 (Synonym: BMH-23)** [67]	SF767 cell WCE incision assay	Fluorescence intercalation displacement assay; no binding	Did not affect AP-1 DNA binding in vitro	NA	NA

* Note: WCE—whole cell extract; NA—data not available.

**Table 2:** Major Ape1 redox inhibitors and their characteristics.

Inhibitor Name	Transcription Factor Target			Ape1 Endo Activity	
	NF-kB	AP-1	HIF-1a		
**APX2009** [68,69]	Transactivation in a cell-based reporter assay system	Electrophoretic mobility shift assay (EMSA *)	NA *	In vitro AP site cleavage increased	
**E3330 (APX3330)** [23,70]	Transactivation in a cell-based reporter assay system and EMSA	Transactivation in a cell-based reporter assay system and EMSA	EMSA	In vitro AP site digestion; no effect	
**C10** [71]	NA	EMSA; Inhibited	NA	In vitro AP site digestion; no effect	

* Note: EMSA—electrophoretic mobility shift assay; NA—data not available.

## Data Availability

Data is contained within the article.
